# Double nylon loop-based inner traction technique promoting endoscopic submucosal dissection of a giant pedunculated adenoma in the ascending colon

**DOI:** 10.1055/a-2351-3420

**Published:** 2024-07-15

**Authors:** Fu-qiang Liu, Xiao Hu, Yun-chao Yang, Xiangrong Zhou, Wenjuan Ding, Zhi-qiang Du, Wei-hui Liu

**Affiliations:** 1 546231 Department of Gastroenterology, The Peopleʼs Hospital of Jianyang City, Jianyang, China; 2 Department of Gastroenterology and Hepatology, Sichuan Provincial Peopleʼs Hospital, School of Medicine, University of Electronic Science and Technology of China, Chengdu, China

Endoscopic submucosal dissection (ESD) is feasible for the removal of giant pedunculated polyps with thick stalks when conventional snare resection is difficult 1 2 . Although pretreatment of the stalks can prevent bleeding in thick-pedunculated polyps, some cases simultaneously comprising massive polyp heads often cause restricted operability and poor visibility, thereby increasing operative challenges 3 4 5 . Herein, we describe a novel inner traction technique to facilitate ESD of a large thick-pedunculated polyp by securely ligating and effectively pulling the stalk with double nylon loops ( Video 1 ).

Double nylon loop-based inner traction technique is used to promote endoscopic submucosal dissection of a giant pedunculated adenoma in the ascending colon.Video 1

A 40-year-old patient was referred for endoscopic treatment following colonoscopy confirmation of a pedunculated polyp with a huge head and thick stalk in the ascending colon ( Fig. 1 **a** ). Because the enormous lesion nearly completely blocked the colonic lumen, we proactively performed pre-ligation and pre-traction before ESD. Initially, with the assistance of a foreign body forceps, a nylon loop was securely ligated at the base of the stalk to limit bleeding ( Fig. 1 **b** ). Next, another nylon loop was secured under the head of the lesion, and its end was precisely anchored to the opposite intestinal wall to attain inner traction ( Fig. 1 **c** ). The inner traction device improved operability and visibility by effectively straightening and exposing the thick stalk, facilitating quick dissection of the stalk between the two ligated loops, thus enabling minimizing bleeding ( Fig. 1 **d** ). Finally, several clips were placed circumferentially to reinforce the ligated loop, preventing loop slippage-related bleeding post-resection ( Fig. 1 **e** ). The specimen was extracted and presented with a normal boundary ( Fig. 1 **f** ). Histological analysis confirmed the polyp as a villous adenoma with complete resection.

**Fig. 1:**
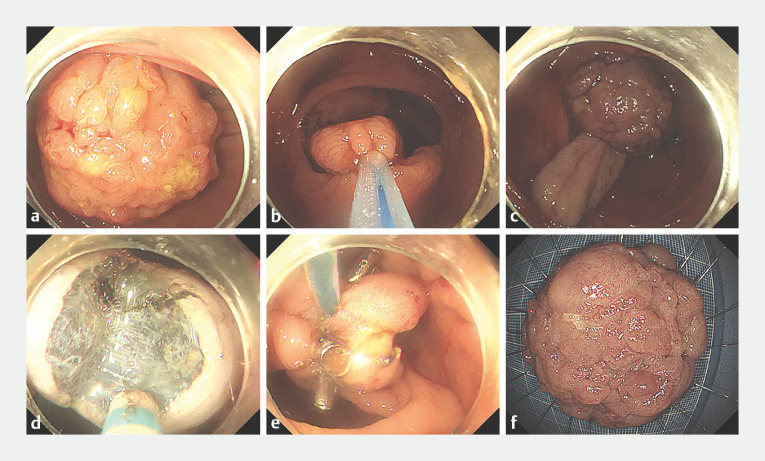
Innovative double nylon loop-based inner traction technique facilitating endoscopic submucosal dissection of a giant pedunculated polyp. **a** A giant ascending colon polyp with a sizable head and extremely thick stalk. **b** A nylon loop was ligated at the base of the stalk to minimize bleeding. **c** Another nylon loop was fastened under the head of the lesion and the end of loop was properly fixed on the opposite intestinal wall to allow inner traction. **d** Dissection of the stalk between the two ligated nylon loops. **e** The ligated nylon loop was well secured by the circumferential placement of clips. **f** The resected specimen measured 5.0 × 4.0 cm with enough base.

The innovative double nylon loop-based inner traction technique should become a priority strategy for endoscopic resection of giant thick-pedunculated polyps because it could reliably ligate the thick stalk pre-resection to minimize bleeding and efficiently expose the stalk to safely and conveniently resect the whole lesion.

Endoscopy_UCTN_Code_TTT_1AO_2AG_3AD

## Notes

Endoscopy E-Videos https://eref.thieme.de/e-videos
*E-Videos* is an open access online section of the journal *Endoscopy* , reporting on interesting cases and new techniques in gastroenterological endoscopy. All papers include a high-quality video and are published with a Creative Commons CC-BY license. Endoscopy E-Videos qualify for HINARI discounts and waivers and eligibility is automatically checked during the submission process. We grant 100% waivers to articles whose corresponding authors are based in Group A countries and 50% waivers to those who are based in Group B countries as classified by Research4Life (see: https://www.research4life.org/access/eligibility/ ). This section has its own submission website at https://mc.manuscriptcentral.com/e-videos .
